# Assessment of radiographers’ competences from the perspectives of radiographers and radiologists: a cross-sectional survey in Lithuania

**DOI:** 10.1186/s12909-017-0863-x

**Published:** 2017-01-26

**Authors:** Aurika Vanckavičienė, Jūratė Macijauskienė, Aurelija Blaževičienė, Algidas Basevičius, Bodil T. Andersson

**Affiliations:** 1 Department of Nursing and Care, Lithuanian University of Health Sciences, Medical Academy, Eivenių str. 2, LT-50009 Kaunas, Lithuania; 2 Faculty of Nursing, Lithuanian University of Health Sciences, Medical Academy, Kaunas, Lithuania; 3 Department of Radiology, Lithuanian University of Health Sciences, Medical Academy, Kaunas, Lithuania; 4 Department of Health Sciences, Faculty of Medicine, Lund University, Lund, Sweden

**Keywords:** Radiography, Competence, Assessment

## Abstract

**Background:**

Assessing radiographers’ clinical competence is of major importance in all medical imaging departments, and is a fundamental prerequisite for guaranteeing professional standards in both nursing care and radiography. Despite the fact that self-assessment has been reported to be the most common form of competence evaluation only several studies defining the radiographers’ self-assessment of clinical competencies were identified. The aim of the study was to evaluate radiographers’ professional competence from the perspectives of radiographers and radiologists by applying the Radiographers’ Competence Scale (RCS).

**Methods:**

The study was conducted in university hospitals of Lithuania. We used the original instrument designed by Swedish researchers – the Radiographers’ Competence Scale (RCS) consisting of two domains: A “Nurse-initiated care” and B “Technical and radiographic processes”. The study involved in all 397 respondents; radiographers (250) and radiologists (147) working in departments of diagnostic radiology. Each competence was evaluated twice – the level on a 10-point scale, and the frequency of practical application on a 6-point scale.

**Results:**

The overall level of the radiographers’ competence and the frequency of its use in practice were evaluated high or very high by both respondent groups. The radiographers attributed the highest evaluations to such competences as “Encouraging and supporting the patient” and “Collaborating with other radiographers”, while the lowest evaluations were attributed to “Guiding the patient’s relatives” and “Empowering the patient by involving him/her in the examination and treatment” competences. The radiologists attributed the highest evaluations to such competences as “Collaborating with radiologists” and “Independent carrying out of the radiologist’s prescriptions”, while the lowest evaluations – to the same competences as the radiographers did. Irrespectively of the work experience and age, the radiographers gave significantly higher ratings to all competences that the radiologists did (*p* < 0.001).

**Conclusions:**

Both groups of the respondents attributed high or very high evaluations to the competences in both the “Nurse-initiated care” and the “Technical and radiographic processes” domains.

## Background

The science and practice of radiography emerged over a century ago, yet it still raises much discussion concerning the role of a radiographer and the limits of his/her competence, independence, and responsibility when carrying out medical imaging procedures [1–3]. The science of radiography developed differently in each European country, which resulted in significant differences in the professional education and training of these specialists [4, 5]. These healthcare specialists organize their workplaces, plan, carry out, and evaluate diagnostic and therapeutic radiological procedures according to the defined competences, ensure the quality of the provided medical radiological procedures and the safety of the patients and other people during those procedures, and also cooperate with other healthcare specialists as well as professionals of other fields [3, 6, 7]. However, during the recent years, the very rapid development of the diagnostic and therapeutic imaging modalities, the continuous emergence of new examination techniques, and the increasing volume and complexity of the services has caused changes and expansion in the fields and the character of radiographers’ professional activity. As a result, their daily work requires increasing amounts of professional knowledge that is not only technical in nature, but is also related to patient care [8, 9]. Radiographers spend increasing amounts of time working with information technologies or performing nursing-related functions associated with patient care rather than focusing solely on medical imaging procedures or administrative functions [10–13]. Radiographers are responsible for the patients’ physical, psychological, and social well-being during the radiography procedures, and their competence directly influences the quality of the procedures as well as patient safety and care during these procedures [1, 9].

In Lithuania, the profession and the fields of activity of a radiographer have been under-documented, and no competence studies have been conducted so far. Therefore, this study is timely and relevant because knowledge about and the analysis of one’s profession promote progress, recognition, and autonomy of this profession, as well as the concordance with the competences of radiographers from other EU countries [7, 14–18].

## Objectives

The aim of the study was to evaluate radiographers’ professional competence from the perspectives of radiographers and radiologists by applying the Radiographers’ Competence Scale (RCS).

## Methods

### Participants

Two groups of respondents were invited to this cross-sectional study - radiographers (271) and radiologists (172) from five Lithuanian university hospitals, who were working during the studied period. These five hospitals the only structures providing all types of medical imaging examinations in Lithuania. The studied population consisted of radiographers and radiologists working together and providing services in diagnostic radiology, including X-ray, computed tomography, nuclear medicine, magnetic resonance imaging, and angiography.

According to the Personnel Report of the Health Information Center of the Institute of Hygiene (HI SIC), in total, there were 865 radiographers and 375 radiologists in Lithuania in 2014.

### Data collection

In this study, we used an instrument designed by a Swedish researcher Bodil T. Andersson - the *Radiographers’ Competence Scale* (RCS) [18].

The pre-established *Radiographers’ Competence Scale* (RCS) used in the questionnaire survey was taken from an original validated survey - Bodil T. Andersson’s - the *Radiographers’ Competence Scale* (RCS) [18], and was modified after expert opinion and suggestions from radiographers in order to be most influential in the radiography setting. A translated and validated Lithuanian version following the technique of inverse translation was used in this study [19]. This is the only instrument for the evaluation of radiographers’ professional competence that we could find in scientific databases. The questionnaire consists of two domains: A. - the evaluation of 18 competences related to “Nurse-initiated care”, and B. - the evaluation of 10 competences related to “Technical and radiographic processes”. The Cronbach’s alpha of the questionnaire is 0.87 [20].

Considering the fact that the study included two groups of respondents, two variants of the questionnaire were prepared: one – for the radiographers (self-assessed level of competences and use in practice), and one – for the radiologists (to assess the radiographers’ competence level from their point of view). In total, the respondents evaluated 30 competences.

The respondents evaluated each competence twice. First, they evaluated the level of the competences on a 10-point scale, (1 point indicating the lowest evaluation, and 10 points – the highest). Subsequently, they evaluated the frequency of the practical application of the competence by marking the suitable evaluation using a 6-point Likert scale, where 1 point meant - “never” and 6 points - “always”. These questions allowed for evaluating the respondents’ attitude towards the level of the competences and the frequency of their use in practice.

### Ethical considerations

This study was conducted in accordance with the principles outlined in the Declaration of Helsinki. Acquiring a permission from the Lithuanian Bioethics Committee was not necessary for this study. The permission to carry out the research was obtained from heads of the hospitals involved in the study. Permission to use the RCS was received from the author of the instrument - Bodil T. Andersson (Faculty of Medicine, Department of Health Sciences, Lund University).

Confidentiality of respondents was assured. Anonymity was maintained, as respondents were never asked for their names, surnames, or addresses. The collected data were summarized and reported in the aggregate and used only for scientific purposes. Radiographers had the right to refuse participation in the study or withdraw at any point without penalty. The methodology of the survey was applied with minimal risk of harm to the study participants.

### Data analysis

Statistical data analysis was conducted by applying the statistical data storage and analysis software package SPSS v. 19 [21]. The level of significance selected for testing data points was established at *p* ≤ 0.05, meaning that the difference was statistically significant. Descriptive statistics was used to calculate the mean values of the variables within a 95% confidence interval. The standard deviation was used to describe the spread of the values. A statistical analysis of qualitative ordinal variables was carried out by applying the chi-square (*χ* ^2^) test, and degrees of freedom (*df*) ware calculated. The non-parametric Mann–Whitney test and its z value were used for the proportional comparison between two groups, while the non-parametric Kruskal-Wallis test was used for the proportional comparison of more than two groups. Spearman’s correlation coefficient (*r*) was used to determine the degree of dependence between the subjects’ level of competence and the demographic characteristics, the frequency of the application of the elements of competences, and the relationship between practical experience and the subjects’ age.

## Results

### Sample characteristics

Data collection took place during April 2014. The response rate among radiographers was 93%, and among radiologists – 85%. Only fully completed questionnaires (250 from radiographers, and 147 - from radiologists) were included into the analysis. After the translation, the reliability of the Lithuanian version of the questionnaire was evaluated by calculating Cronbach’s alpha coefficient for each domain of the questionnaire, indicating the internal consistency of the scale. Cronbach’s alpha for domain A of the questionnaire, was 0.95 and for domain B - 0.95. Cronbach’s alpha for the whole instrument was 0.97.

The respondents who participated in the survey were distributed into four groups according to their work experience. Among radiographers, the predominant respondents were women with over 25 years of work experience who were working in the field of diagnostics with diagnostic radiology or computed tomography equipment (Table 1). The median age (min-max; mean) was 50 years (23–73; 48.2). Statistically significant differences were found between the age and the work experience groups.

**Table 1:** Socio-demographic characteristics of the respondents

	Radiographers	Radiologists
	Total	Total
	n (%)	n (%)
	250 (100.0)	147 (100.0)
Age, median (min-max; mean)	50 (23–73; 48)	43 (29–75; 44)
Gender, n (%)	n (%)	n (%)
Women	237 (94.8)	96 (65.3)
Men	13 (5.2)	51 (34.7)
Work experience	n (%)	n (%)
0–5 years (I group)	44 (17.6)	40 (27.2)
> 5–15 years (II group)	73 (29.2)	36 (24.5)
> 15–25 years (III group)	53 (21.2)	31 (21.1)
> 25 years (IV) group	80 (32)	40 (27.2)
Field of work (department), n (%)	n (%)	n (%)
Diagnostic radiology	200 (80.0)	118 (80.3)
Nuclear medicine	26 (10.4)	16 (10.9)
Interventional radiology/angiography	22 (8.8)	13 (8.8)

The radiologists who participated in the survey were somewhat younger; with respect to the work experience, their distribution in the groups of 0–5 years and over 25 years of work experience was equal. In this group of subjects, radiologists working in the field of diagnostics predominated as well.

### Assessed level of professional competences

The radiographers presented rather high evaluations of the majority of competences in both domains. The median (min-max; mean) of the mean evaluations of competences in dimension A “Nurse-initiated care” was 9.0 (5.5-10.0; 8.86), and the median (min-max; mean) of the mean evaluations of competences in dimension B “Technical and radiographic processes’ – 9.0 (2.5-10.0; 8.82). The radiologists presented somewhat lower medians of the mean evaluations of radiographers’ competences in both dimensions than radiographers did (*p* < 0.001) (Table 2).

**Table 2:** The comparison of the evaluation of radiographers’ competences

Radiographers’ competences	Radiographers	Radiologists
	Median (min-max; mean)	
A. Competences related to ”Nurse-initiated care”^a^		
Mean evaluation	9.0 (5.5-10.0; 8.86)	8.05 (3.7-10.0; 7.93)
B. Competences related to “Technical and radiographic processes”^a^		
Mean evaluation	9.0 (2.5-10.0; 8.82)	8.10 (3.5-10.0; 7.93)

^a^The Mann Whitney test. The evaluations of all attributes differed statistically significantly (*p* < 0.001)

Both radiographers and radiologists presented the highest evaluations of the competences “Encouraging and supporting the patient”, “Collaborating with other radiographers”, and “Collaborating with radiologists”, while the competences “Guiding the patient’s relatives” and “Empowering the patient by involving him/her in the examination and treatment” received the lowest evaluations.

Table 3 illustrates that all four work experience groups rated their professional competencies as high. The levels increased in line with the number of years in the current position. In the dimension “Nurse-initiated care”, the radiographers with over 25 years of work experience gave better evaluations of such competences as “Independent carrying out of the specialist physician’s prescriptions” (*p* < 0.001), “Independent carrying out of the radiologist’s prescriptions” (*p* = 0.005), “Guiding the patient’s relatives” (*p* = 0.017), and “Disseminating professional experience” (*p* = 0.004), compared to radiographers with 5 or fewer years of experience. Radiographers with longer work experience also provided better ratings of such competences as “Adequately informing the patient”, “Collaborating with other radiographers”, and “Participating in qualification improvement courses and self-development” than their less experienced colleagues did (Table 3).

**Table 3:** Radiographers’ self -assessed competences level with respect to their work experience

	Radiologists mean evaluations	Radiographers mean evaluations	0–5 years (I)	>5–15 years (II)	>15–25 years (III)	>25 years (IV)	*p*-value	Pairwise comparison*
			Median (min-max; mean)					
A. Competences related to “Nurse-initiated care”								
Independent carrying out the specialist’s prescriptions	7.66	8.49	8 (1–10; 7.75)	9 (1–10; 8.58)	8 (1–10; 8.36)	9 (4–10; 8.90)	0.001	C
Independent carrying out the radiologist’s prescriptions	8.30	8.86	9 (5–10; 8.32)	9 (2–10;8.81)	9 (4–10; 9.08)	9 (2–10; 9.05)	0.006	B; C
Adequately informing the patient	8.15	9.20	9 (5–10; 9.00)	10 (7–10; 9.36)	9 (7–10; 9.04)	10 (5–10; 9.28)	0.027	A
Guiding the patient’s relatives	7.09	8.10	7 (1–10; 7.57)	9 (1–10; 8.44)	8 (1–10; 7.57)	9 (2–10; 8.45)	0.001	A; C; D; F
Collaborating with other radiographers	8.36	9.29	10 (5–10; 9.16)	9 (7–10; 9.14)	10 (6–10; 9.51)	10 (4–10; 9.36)	0.033	D
Disseminating professional experience	7.87	8.78	8 (4–10; 8.25)	9 (6–10; 9.95)	9 (4–10; 8.72)	9 (3–10; 8.96)	0.004	A; C
Participating in quality improvement regarding patient safety and care	7.50	8.62	9 (1–10; 8.41)	9 (6–10; 9.15)	9 (1–10; 8.32)	9,5 (3–10; 9.14)	0.005	F
B. Competences related to “Technical and radiographic processes”								
Producing accurate and correct images	8.10	9.04	9 (7–10; 8.84)	9 (1–10; 9.08)	9 (5–10; 8.81)	10 (1–10; 9.26)	<0.001	C; F
Evaluating the quality of the medical image in relation to the referral and the question stated therein	7.86	8.62	8 (5–10; 8.39)	9 (1–10; 8.89)	9 (5–10; 8.43)	9 (1–10; 8.63)	0.018	A
Optimizing the quality of the image	7.52	8.63	8 (5–10; 8.25)	9 (1–10; 8.89)	8 (5–10; 8.53)	9 (1–10; 8.66)	0.027	A
Preliminary assessment of images	7.52	8.24	8 (4–10; 7.66)	9 (3–10; 8.34)	8 (1–10; 8.21)	9 (2–10; 8.50)	0.006	A; C

*p-value of the Kruskal-Wallis criterion (A. I-II; B. I-III; C. I-IV; D. II-III; E. II-IV; F. III-IV - *p*-values of pairwise comparison)

Concerning competences related to the “Technical and radiographic processes”, the radiographers scored the presented evaluations of such competences as “Responsibility for preparing the medico-technical equipment” - 10.0 (5–10; 9.42) and “Producing accurate and correct images” - 9.0 (1–10; 9.04), while the lowest evaluation scored “Preliminary assessment of images” - 8.0 (1–10; 8.24) competence.

Radiographers with over 25 years of work experience presented higher ratings of the competences “Producing accurate and correct images” and “Preliminary assessment of images” than their colleagues with fewer than 5 years of experience did (*p* = 0.001) (Table 3).

### Self-assessed use of professional competences

The majority of the radiographers stated that they almost always used competences related to the “Nurse-initiated care” domain in their daily professional practice. They almost always used the competences “Observing and monitoring the patient” (91.2%), “Collaborating with radiologists” (90.8%), and “Adequately informing the patient” (89.2%), yet only slightly more than one-half of them applied “Empowering the patient by involving him/her in the examination and treatment” (58.8%).

The pairwise comparison of the competence use of the domain A “Nurse-initiated care” showed that compared with their less experienced colleagues, radiographers with greater work experience more frequently used such competences as “Guiding the patient’s relatives”, “Observing and monitoring the patient”, “Collaborating with radiologists”, and “Disseminating professional experience” (*p* < 0.001) (Table 4).

**Table 4:** Radiographers self-assessed use of competences related to the “Nurse-initiated care”

A. Competences related to the “Nurse-initiated care”	Rate of application^a^	Overall evaluation, %	0-5 years (I)	>5-15 years (II)	>15-25 years (III)	>25 years (IV)	*p*-value	Pairwise comparison**
Independent carrying out the specialist’s prescriptions	1–3	16.8	18.2	17.8	11.3	18.8	0.042	
	4	15.6	20.5	16.4	26.4	5		C
	5–6	67.6	61.4	65.8	62.3	76.3		
Adequately informing the patient	1–3	3.2	4.5	2.7	1.9	3.8	0.004	
	4	7.6	15.9	1.4	17	2.5		F
	5–6	89.2	79.5	95.9	81.1	93.8		A
Guiding and educating the patient	1–3	13.6	20.5	2.7	22.6	13.8	0.017	A
	4	15.6	18.2	20.5	7.5	15		
	5–6	70.8	61.4	76.7	69.8	71.3		
Empowering the patient by involving him/her in the examination and treatment	1–3	20	27.3	11	18.9	25	0.027	A
	4	14.8	13.6	12.3	26.4	10		D
	5–6	65.2	59.1	76.7	54.7	65		
Guiding the patient’s relatives	1–3	34	52.3	31.5	46.3	18.8	<0.001	
	4	7.2	11.4	8.2	9.4	2.5		
	5–6	58.8	36.4	60.3	45.3	78.8		F
Encouraging and supporting the patient	1–3	8.4	18.2	5.5	11.3	3.8	0.025	C
	4	8.8	13.6	12.3	5.7	5		
	5–6	82.8	68.2	82.2	83	91.3		C
Observing and monitoring the patient	1–3	1.2	4.5	1.4	0	0	<0.001	
	4	7.6	25	5.5	3.8	2.5		
	5–6	91.2	70.5	93.2	96.2	97.5		A; C
Collaborating with other radiographers	1–3	5.2	15.9	5.5	3.8	0	0.001	
	4	6.8	4.5	13.7	3.8	3.8		
	5–6	88	79.5	80.8	92.5	96.3		E
Collaborating with radiologists	1–3	6.4	27.3	1.4	1.9	2.5	<0.001	
	4	2.8	2.3	2.7	7.5	0		
	5–6	90.8	70.5	95.9	90.6	97.5		A
Supervising and training colleagues and other co-workers	1–3	26	45.5	21.9	22.6	21.3	0.031	A
	4	10.4	9.1	12.3	15.1	6.3		
	5–6	63.6	45.5	65.8	62.3	72.5		C
Disseminating professional experience	1–3	15.6	34.1	11	22.8	5	0.001	A; F
	4	16.8	11.4	15.1	15.1	22.5		
	5–6	67.6	54.5	74	62.3	72.5		
Participating in refresher courses	1–3	9.2	9.1	6.8	20.8	3.8	0.010	
	4	13.6	18.2	11	18.9	10		
	5–6	77.2	72.7	82.2	60.4	86.3		D
Participating in quality improvement regarding patient safety and care	1–3	19.2	29.5	15.1	9.4	23.8	0.012	
	4	7.6	15.9	8.2	7.5	2.5		
	5–6	73.2	54.5	76.7	83	73.8		B

^a^evaluation of the application of the competence in professional activity: 1–3 sometimes; 4 often; 5–6 almost always

**p-value of the Chi criterion (A. I-II; B. I-III; C. I-IV; D. II-III; E. II-IV; F. III-IV – *p*-values of pairwise comparison)

The evaluation of the frequency of the application of competences related to the domain B. “Technical and radiographic processes” showed that radiographers almost always assumed responsibility for preparing the medico-technical equipment (91.2%), for producing accurate and correct images (89.6%), and for minimizing radiation doses for the patient and the staff (82.8%), yet almost one-fourth of them only sometimes performed preliminary assessment of the images (Table 5).

**Table 5:** Radiographers self-assessed use of competences related to the “Technical and Radiographic Processes”

B. Competences related to the “Technical and Radiographic Processes”	Rate of application^a^	Overall evaluation, %	0–5 years (I)	>5–15 years (II)	>15–25 years (III)	>25 years (IV)	*p*-value	Pairwise comparison^a^
Organizing and planning taking account of the clinical situation	1–3	10.8	20.5	1.4	11.3	13.8	0.014	A
	4	10.4	11.4	8.2	17	7.5		
	5–6	78.8	68.2	90.4	71.7	78.8		A
Responsibility for preparing the medico-technical equipment	1–3	1.2	4.5	0	1.9	0	0.011	
	4	7.6	11.4	1.4	15.1	6.3		
	5–6	91.2	84.1	98.6	83	93.6		A
Prioritizing patients in the work flow	1–3	8.8	18.2	5.5	7.5	7.5	0.023	
	4	14.8	2.3	16.4	11.3	22.5		C
	5–6	76.4	79.5	78.1	81.1	70		
Adapting the examination to the patient’s prerequisites and needs	1–3	8.8	9.1	12.3	3.8	8.8	0.007	
	4	14	9.1	4.1	28.3	16.3		D
	5–6	77.2	81.8	83.6	67.9	75		
Minimizing radiation doses for patient and staff	1–3	6	13.6	5.5	0	6.3	0.014	
	4	11.2	4.5	5.5	15.1	17.5		
	5–6	82.8	81.8	89	84.9	76.3		
Optimizing the quality of the image	1–3	8.8	4.5	2.7	22.6	7.5	0.001	D
	4	10.4	13.6	4.1	9.4	15		
	5–6	80.8	81.8	93.2	67.9	77.5		D
Preliminary assessment of images	1–3	18.4	29.5	19.2	18.9	11.3	0.015	
	4	16.8	15.9	8.2	28.3	17.5		D
	5–6	64.8	54.5	72.6	52.8	71.3		

^a^evaluation of the application of the competence in professional activity: 1–3 sometimes; 4 often; 5–6 almost always

*p*-value of the Chi criterion (A. I-II; B. I-III; C. I-IV; D. II-III; E. II-IV; F. III-IV - values of pairwise comparison)

The pairwise comparison of the competences in domain B. “Technical and radiographic processes” revealed an association between work experience and the frequency of the application of competences in professional practice. An especially significant statistical association was found between work experience and the practical use of the competences “Organizing and planning taking account of the clinical situation” and “Optimizing the quality of the image”. Radiographers with greater work experience more frequently applied these competences in practice than their less experienced colleagues did (*p* < 0.001) (Table 5).

### Variables associated with professional competences

The analysis of the association between the mean evaluation of the competences from the “Nurse-initiated care” and the “Technical and radiographic processes” domains and the radiographers’ age and work experience revealed a weak yet statistically significant relationship: with age – respectively, *r* = 0.146 and *r* = 0.179, and with work experience – respectively, *r* = 0.109, and *r* = 0.114,

A weak yet statistically significant relationship was found between the frequency of the applications of competences from both domains and the respondents’ age (*r* = 0.134).

Concerning the radiologists’ opinion about radiographers’ competence, their mean evaluations of competences from the “Nurse-initiated care” domain had a weak yet statistically significant relationship with the respondents’ age only (*r* = 0.174), while the mean evaluations of the competences from the “Technical and radiographic processes” statistically significantly correlated with the respondents’ age and work experience – respectively, *r* = 0.207 and *r* = 0.18.

## Discussion

The radiographer’s abilities and competences are vital for the patient. As one of the most advanced areas of medicine, the continuously changing and developing field of diagnostic radiology directly influences radiographers’ competence and increases the demand for very highly qualified personnel.

Undoubtedly, the development of technical procedure-related competences is the main focus in a radiographer’s professional activity [8–12, 22, 23]. The content of the specialist education and training programs is also more oriented towards technical subjects [3, 4, 17, 24]. However, patient care is obviously an integral part of a radiographer’s activity [1, 2, 20]. Cooperation with the patient prior to, during, and after the procedure is the activity that directly reflects a radiographer’s nursing skills [25]. The results of a qualitative study conducted in Sweden showed that a radiographer’s professional activity and competences may be differentiated into those directly or indirectly associated with patient care. In this study, were analyzed fields of a radiographer’s activity that are directly related to patient care – informing and guiding the patient, use of protective measures, injection of medications, adaptation of the procedure to the patient’s needs, protection of the patient’s integrity, monitoring during the procedure, and responding to acute reactions.

The results of our study also showed that radiographers attributed high or very high evaluations to competences from both the “Nurse-initiated care” and the “Technical and radiographic processes” domains, and no statistically significant difference in the mean evaluations was observed. The evaluation of the application of the analyzed competences showed that Lithuanian radiographers often or almost always applied the majority of the patient care-related competences listed in the questionnaire.

During daily practice, a radiographer always works in a team with a radiologist and plans and carries out medical imaging procedures according to the radiologist’s prescriptions. Thus, radiologists’ opinion was very important to us in order to ensure feedback and for a more objective evaluation and disclosure of attitudes towards radiographers’ competences. Radiologists presented high to very high evaluations of radiographers’ competences, yet their evaluations of all competences were lower compared to those presented by radiographers (*p* < 0,001). Both radiologists and radiographers gave their highest or lowest evaluations for virtually the same competences. It is likely that such results were due to the work organization principles, competences, limits of responsibility, or a lack of time. In many cases, the time a radiographer can allocate to a single patient is short, and it is likely that in cases of a rapid pace of work, the competence “Guiding the patient’s relatives” is rarely applied – and thus receives low evaluations. However, the competences that received high evaluations from both groups of the respondents – “Observing and monitoring the patient”, “Encouraging and supporting the patient”, and “Collaborating with radiologists” – were directly related to patient care and the quality of the procedures.

The competence “Preliminary assessment of images” also received the lowest evaluations from both respondent groups because in Lithuania, like in many countries, the responsibility for the evaluation of radiographs lies with radiologists. High evaluations of the competences “Responsibility for preparing the medico-technical equipment”, “Adapting the examination to the patient’s prerequisites and needs”, and “Producing accurate and correct images” indicate a high level of professionalism, and high evaluations of the competence “Prioritizing patients in the work flow” indicate good work planning and management skills.

As in many other evaluations of competences in various occupational groups, our study also revealed a link between the evaluation of the competence and the frequency of its practical application and the evaluators’ age and work experience [20, 25–28].

Concerning human resources in healthcare, nurses’ competence has been probably researched the most, and its measurements and evaluations have been carried out in various aspects, using the Nurse Competence Scale [29, 30]. Only a few sources on radiographers’ competence have been found in scientific literature [2, 9, 20, 22, 25]. The Swedish study on radiographers’ competence is one of the most recent and comprehensive studies in this area, focusing on radiographers’ professional competence in the field of diagnostics [20, 25]. The instrument of this study was (with the author’s consent) also used in Lithuania.

### Study limitations

The weak side of this study is that the evaluation of radiographers’ competence, the application of the general instrument, and the comparison of the obtained results are complicated due to certain differences in work organization principles and specialist education between different countries, which results in differing limits of responsibility and independence. The validation process and reliability assessment of the RCS is not complete in this study, as there is no validated and approved Lithuanian version of the RCS that could be used for referencing. In Lithuania, radiographers are not licensed for healthcare activity, and there is no Medical Norm that would clearly and precisely define a radiographer’s competence and the limits of responsibility and independence in the areas of his/her professional activity. Another limitation of the study is that it was only conducted in university hospitals where radiographers are likely to have higher and broader competence and apply it more frequently in their practice. In addition, self-assessment is subjective evaluation and has no definitive criteria allowing the participants to scale their competences accurately, and thus the evaluations could wary from person to person. On the other hand, radiologists may also have difficulty in assessing radiographers’ competence levels, because they have to evaluate the whole occupational group rather than individual radiographer. Arguably, radiographers’ competence levels may differ and constantly may vary, so it might have a slight impact on the results of the study.

### Implication for practice

Self-evaluation of their competence will allow Lithuanian radiographers to review their knowledge and abilities and to reflect on their professional behavior with patients and colleagues. Systematic and repeated studies on competence would undoubtedly stimulate the development and continuous improvement of the profession, which, in turn, would improve patient nursing and care. Conducting this study across multiple European countries would allow for the comparison of the results in the international context, and would reveal the similarities and differences in the professional activity of radiographers across different countries. The results of the study would also be useful for heads of healthcare institutions as well as for adjusting occupational standards, medical norms, and other documents regulating radiographers’ professional activity on both the national and the international levels. Educational institutions engaged in professional education and training of radiographers may use the results of this study for the adjustment of their curricula and expected learning outcomes.

## Conclusion

Both groups of the respondents – radiographers and radiologists - gave high or very high evaluations of radiographers’ competences and the frequency of their application in practice. The competences from both the “Nurse-initiated care” and the “Technical and radiographic processes” domains were equally important components of radiographers’ professional activity. The results of the study suggest that the radiographers’ work experience and age were directly related to the evaluation of the competences as well as to their practical application.

## Abbreviations

RCS: Radiographers competence scale
